# Effects of Circadian Phase Tailored Light Therapy on Sleep, Mood, and Cognition in Alzheimer’s Disease: Preliminary Findings in a Pivotal Study

**DOI:** 10.3389/fphys.2021.755322

**Published:** 2022-01-06

**Authors:** Riccardo Cremascoli, Davide Sparasci, Gianluca Giusti, Stefania Cattaldo, Elisa Prina, Fausto Roveta, Francesco Bruno, Cristina Ghezzi, Silvia Cerri, Marta Picascia, Sara Bernini, Elena Sinforiani, Michele Terzaghi, Lorenzo Priano, Alessandro Mauro, Raffaele Manni

**Affiliations:** ^1^Department of Brain and Behavioural Sciences, University of Pavia, Pavia, Italy; ^2^Unit of Sleep Medicine and Epilepsy, IRCCS Mondino Foundation, Pavia, Italy; ^3^Department of Neurology and Neurorehabilitation, Istituto Auxologico Italiano, IRCCS, San Giuseppe Hospital, Piancavallo, Italy; ^4^Department of Neurosciences “Rita Levi Montalcini”, University of Torino, Turin, Italy; ^5^Laboratory of Clinical Neurobiology, Istituto Auxologico Italiano, IRCCS, San Giuseppe Hospital, Piancavallo, Italy; ^6^Laboratory of Functional Neurochemistry, IRCCS Mondino Foundation, Pavia, Italy; ^7^Neuropsychology/Alzheimer’s Disease Assessment Unit, IRCCS Mondino Foundation, Pavia, Italy

**Keywords:** circadian rhythm, melatonin, light therapy, sleep quality, cognition, Alzheimer’s disease, dim light melatonin onset (DLMO)

## Abstract

It is shown that the circadian system is affected in patients with Alzheimer’s disease (AD) even at an early stage of the disease and that such dysfunction may be detrimental to sleep, mood, and cognitive functioning. Light is a strong central modulator of the circadian rhythms and is potentially beneficial to mood and cognitive functioning *via* a direct effect or indirectly *via* its modulating effects on circadian rhythms. This study focuses on tracking the effect of light therapy on sleep quality, mood, and cognition in AD of mild/moderate severity. We performed a single-blind randomized controlled trial to investigate the effects of a light therapy treatment tailored to the individual circadian phase as measured by dim light melatonin onset (DLMO). Such a treatment induced an objective circadian phase shift consistent with the melatonin phase response curve to light exposure, led to a shortening of the phase angle DLMO-falling asleep time, and was associated with an improvement in subjective sleep quality and cognitive performance.

## Introduction

Recent studies showed that circadian system dysfunction played a role in the genesis of sleep disorders in Alzheimer’s disease (AD) and may have been detrimental to cognitive functioning (Saeed and Abbott, 2017). Changes in circadian rhythmicity were associated with reduced quality of nocturnal sleep, increased daytime sleepiness, and reduced cognitive performance (Reid et al., 2011; Wright et al., 2012). There is evidence in the literature that light therapy is efficacious in the resynchronization of melatonin secretion to the dark-light cycle (Tähkämö et al., 2019). However, evidence is limited to support its use in the treatment of sleep disturbances and agitation in persons with cognitive impairment.

Positive effects of different light therapy protocols on at least one sleep measure were reported in AD (O’Caoimh et al., 2019). However, light therapy, such as bright light therapy, did not show significant effects on cognition, activities of daily life that were included in the activities of daily living scale, sleep-wake disturbances, challenging behavior, or psychiatric disturbances. These were considered in recent systematic reviews and meta-analyses, such as a Cochrane review that specifically examined the results of randomized controlled trials (RCTs) (Forbes et al., 2014; Abraha et al., 2017; Mitolo et al., 2018; Hampton, 2019; Missotten et al., 2019). It is of note that, in the different light therapy protocols that have been considered in AD, light exposure timing was not tailored to the individual circadian phase. However, light treatment should likely be set to the circadian phase. This was suggested by the results of studies that showed that the patterns of 24-h melatonin secretion were irregular in patients with AD (Mishima et al., 1999; Skene and Swaab, 2003). Furthermore, in a previous study, we found that the time of dim light melatonin onset (DLMO) varied in patients who were at an early stage of AD and tended to occur later in the day than in controls (Manni et al., 2019).

In this study, we aimed to evaluate the effects of light treatment on sleep quality, mood, and cognition in patients with mild/moderate AD. The study was based on the notion that setting light exposure according to the circadian phase optimized the efficacy of light as a resynchronizer of melatonin secretion to the dark-light cycle. We performed a single-blind RCT, in which the timing of light exposure was tailored to the circadian phases of patients, as measured by their DLMO time.

## Materials and Methods

### Study Protocol

**FIGURE 1:**
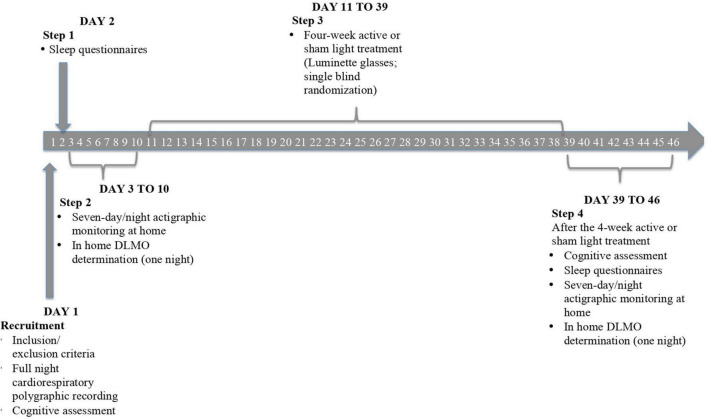
Progressive steps of the study protocol. After the recruitment, all the patients once checked for clinical exclusion criteria, performed a full night cardiorespiratory polygraphic recording, and received the first battery of neuropsychological tests [i.e., Mini-Mental State Examination (MMSE) and Neuropsychiatric Inventory (NPI)] with the addition of Beck’s Depression Inventory. Subsequently, they filled out sleep questionnaires [i.e., Pittsburgh Sleep Quality Index (PSQI) and Epworth Sleepiness Scale (ESS)] under medical supervision. The following step consisted of a 7-day/night actigraphic monitoring through a triaxial actigraphic watch (MotionWatch8), assessing the 24-h activity-rest rhythm and objective sleep parameters of subjects. Salivary melatonin collection was then performed to determine the circadian phase of patients by means of dim light melatonin onset (DLMO). These data permitted us to plan a 4-week tailored light (active or sham) exposure by means of Luminette glasses. At the end of the protocol, we reassessed cognitive, sleep, and circadian measures to evaluate a potentially favorable effect of light delivery.

The study employed a randomized, placebo-controlled, single-blind protocol over a 3-month period. The timeline of the design is displayed in Figure 1.

Participants were recruited at the Neuropsychology/Alzheimer’s Disease Assessment Unit of the Istituto di Ricovero e Cura a Carattere Scientifico (IRCCS, the Scientific Institute for Research, Hospitalization, and Healthcare) Mondino Foundation in Pavia. All the patients had confirmed diagnoses of AD, based on the criteria listed in the *Diagnostic and Statistical Manual of Mental Disorders, Fourth Edition* (DSM-IV) (McKhann et al., 2011).

Patients with AD, who are in an advanced stage of the disease, reportedly lose sensitivity to the synchronization effects of light, due to heavy losses of melanopsin retinal ganglion cells in the retina (Mitolo et al., 2018). Therefore, we recruited only patients with mild/moderate cognitive impairment. We accepted patients who exhibited scores on the Mini-Mental State Examination (MMSE) between 16 and 24, adjusted for age and educational levels (MMSEc).

Exclusion criteria were as follows:

1. Occurrence during a full night of cardiorespiratory polygraph recording of obstructive sleep apnea with an apnea-hypopnea index of >15 and periodic limb movements during sleep at a rate of >10/h;
2. Ocular contraindications: cataract, degenerative macular retinopathy, or narrow-angle glaucoma;
3. Consumption of psychological stimulants, sedatives, antidepressant drugs or melatonin; or
4. Signs of depression. Considering that mood disorders could have influenced sleep and cognition, we evaluated depressive symptoms by means of the Beck’s Depression Inventory both before and after the 4-week light treatment. No patient proved to be affected with major depression.

Participants received the first battery of neuropsychological tests [i.e., the MMSE and the Neuropsychiatric Inventory (NPI) Scale] at the Neuropsychology/Alzheimer’s Disease Assessment Unit of the IRCCS Mondino Foundation. Subjects were subsequently followed at the Unit of Sleep Medicine of the foundation through a four-step experimental protocol.

**Step 1.** The patients completed sleep questionnaires [i.e., the Pittsburgh Sleep Quality Index (PSQI) and the Epworth Sleepiness Scale (ESS)]. The questionnaires were filled out under medical supervision.

**Step 2.** Participants underwent salivary DLMO determination and actigraphic monitoring at home for 7 days/nights. The actigraphic monitoring was performed through the use of a triaxial actigraphic watch (MotionWatch 8), which was equipped with an ambient light sensor. Participants wore the watch for 7 consecutive days and nights on the non-dominant wrist. Patients and caregivers were given 24-h wake-sleep diaries for them to document the sleep habits and behavior of participants during the 7-day period at home. Patients and caregivers were instructed to report off-wrist intervals in the diary.

Bühlmann saliva collection devices were provided to the patients for DLMO determination, in association with written guidance on the procedure for sample collection. Participants and their caregivers were instructed carefully and received specific diaries for them to document the saliva collection times and bedtimes.

The DLMO protocol was planned according to a procedure we previously experienced as reliable and feasible in patients with AD (Pullman et al., 2012; Manni et al., 2019). DLMO was estimated at home in the standard conditions of environmental light (<20 lux), in a sitting posture and during periods of low physical activity, within a 5-h window, and at a saliva sample rate of 60 min. The first sample was taken 3 h before the usual bedtime of a patient. The DLMO time was determined with a fixed threshold of 3 pg/ml salivary melatonin. The estimation of salivary melatonin secretion was provided by means of a Bühlmann enzyme-linked immunosorbent assay (ELISA) kit (EK-DSM), which was designed to measure salivary melatonin levels. This quantitative and highly sensitive test is based on the Kennaway G280 antibody test, which involves a sodium hydroxide pretreatment of the sample followed by neutralization with hydrochloric acid.

**Step 3.** After DLMO determination, participants returned to the sleep unit, where light glasses (Luminette^®^) were provided and a protocol of at-home, 4-week active or sham light treatment was planned.

Patients were randomly assigned to one of the two conditions (i.e., treatment or sham). The randomization list was generated through a simple randomization method that used random number generator software, which was available at www.regione.emilia-romagna.it/sin_info/generatore. The algorithm used at this site coincides with a Lehmer (congruently multiplicative) generator. Allocation concealment is guaranteed by the performance of central randomization at a remote location that is independent of the enrollment site.

The light glasses were a medical device produced by the company Lucimed. They were called Luminette (EAN: 0702382929671), weighed 0.6 kg, and measured 22 × 11 × 11 cm. This device is freely available on the market, and its technical details are available on demand. The device provides blue-enriched light of 10,000 lux perceived. It includes a UV filter and is made to European Certification (CE) standards. The Luminette glasses that were used in this study were set to intensity 3/3 and worn for 20 min/day (Bjorvatn and Pallesen, 2009) for 28 consecutive days. The glasses enabled light exposure to be controlled while good ergonomics were ensured so that the patients could continue with their normal activities.

A controlled exposure was set for the patients in the sham (control) group, through the use of the same Luminette device set at a fluorescence of 50 lux, which is close to ambient light and therefore considered ineffective.

Light delivery was planned according to the individual circadian phase of each participant, as measured by DLMO. Patients were considered to fall into an early, intermediate, or late circadian phase according to DLMO times of before 7.30 p.m., between 7.30 p.m. and 10 p.m., and after 10 p.m., respectively (Pandi-Perumal et al., 2007).

Patients who exhibited late or intermediate circadian phases [patients who were showing a late circadian phase were designated as Late Circadian Phase (LCP) patients (LCPpts)] wore Luminette glasses after spontaneous wake-up in the morning, while those who exhibited an Early Circadian Phase (ECPpts) performed the light therapy 1 h before DLMO. Caregivers, interviewers, and neuropsychologists were blinded to the treatment allocation.

**Step 4**. After completion of the 4-week light treatment, subjects repeated the initial evaluations; all the participants performed neuropsychological tests and filled in sleep questionnaires. Fresh DLMOs were determined to calculate circadian phase shift. A 7-day/night actigraphic monitoring was repeated after active/sham light treatment to evaluate any variation of the primary sleep outcomes.

### Participants

**FIGURE 2:**
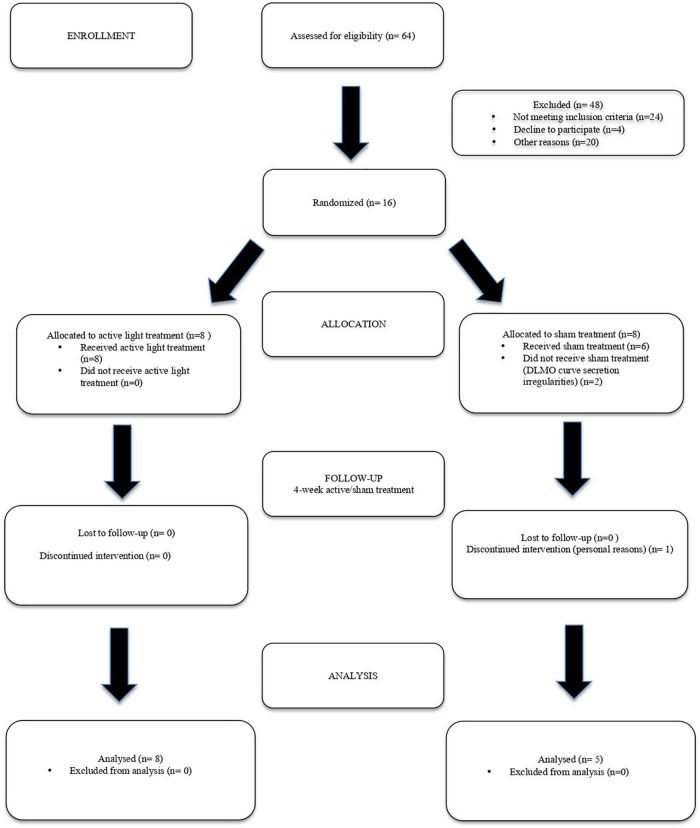
Enrollment process flowchart of participants.

The sample size for this study was calculated according to the indications of the open-source epidemiological statistics for public health.^1^ Based on the literature data and our previous experience, we took as meaningful a difference of 10% in sleep efficiency (SE, primary outcome) before and after Luminette treatment. We decided to verify whether this significant SE improvement would be achieved in each cohort of patients (active vs. sham light treatment). The sample size was calculated according to the following parameters: CI (two-sided) 95%, power 80%, ratio of group sizes 1:1, mean difference 10%, and SD of 13% for both groups. The suggested minimum number of subjects to be enrolled was 54 (27 per group). The enrollment process is shown in Figure 2. Taking into account the possibility that 20% of those asked would leave/refuse, the number of patients to be enrolled was planned to be 32 per group.

However, due to the COVID-19 pandemic, we could select only 40 patients who met the inclusion criteria. Of these, 20 were excluded from the study based on the specified exclusion criteria. Four patients declined the proposal, so only 16 patients could be enrolled.

Eight participants underwent the Luminette intervention protocol, and the other eight, the sham protocol. One subject had to repeat the collection of saliva for melatonin measurement because the quantity of saliva in the first set of cotton swabs was insufficient.

In two members of the sham group, the DLMO at baseline could not be calculated due to irregularities of the secretion curves (“bizarre curves”). We considered a secretion curve to be irregular when the threshold of 3 pg/ml was reached more than one time in an up-and-down secretion pattern. One member of the sham group dropped out before the second actigraphic monitoring could be performed after they had completed the 4-week light delivery and had repeated the DLMO determination. Consequently, the investigation pertained to 13 patients with AD, 8 in the intervention group, and 5 in the sham. The demographic and clinical features of these 13 participants are listed in Table 1 [male/female: 9/4; median age: 73 years; interquartile range (IQR): 70–78]. Patients of both groups were comparable in terms of sex and age. Based on the feedback of caregivers, the compliance rate of Luminette use was nearly 100%. This high rate of compliance could be in part a consequence of the mild stage of AD of the patients who were enrolled.

**TABLE 1:** Demographic and clinical features of patients.

Attribute	Quantity
Participants (number, sex M/F)	13 (9 M/4 F)
Age (median, IQR)	73 (70–78)
MMSEc score (median, IQR)	20.40 (17.70–22.40)
Active light protocol (number, sex, median age, IQR age)	8, 6M/2F, 72 (69–76)
Sham protocol (number, sex, median age, IQR age)	5, 3M/2F, 76 (71–80)
ECPpts in active light protocol (number, sex, median age, IQR age)	2, 1M/1F, 74 (71–76)
LCPpts in active light protocol (number, sex, median age, IQR age)	3, 2M/1F, 73 (71–78)

*ECPpts, Early Circadian Phase patients; LCPpts, Late Circadian Phase patients; MMSEc, Mini-Mental State Examination adjusted for age and educational levels; IQR, interquartile range (quartile 1–quartile 3).*

### Outcome Measures

#### Actigraphic Parameters

This study involved the use of a triaxial actigraphy watch (MotionWatch 8) to measure the sleep-wake rhythms and sleep of participants. The MotionWatch 8 is a wrist-worn device that measures movement activity by means of a piezoelectric accelerometer. Actigraphy is considered a valid and reliable measurement of sleep-wake rhythms and sleep in adults among the general population and has proved to be feasible and reliable in patients with AD (Ancoli-Israel et al., 2003).

The recording mode chosen for this research was the triaxial mode 3. This is an epoch-based recording mode that uses all three of the accelerometer axes to produce a vector magnitude result per epoch. The epoch length for the analysis of data was set at 30 s, the threshold used to analyze the movement was set at 20, and the sampling frequency was set at 50 Hz. During the monitoring phase of the protocol, off-wrist intervals were short and do not influence the analyses.

The actigraphic recording enabled the assessment of the following objective sleep parameters: SE, total sleep time (TST), wake after sleep onset, Sleep Fragmentation Index, mid-sleep time, inter-daily stability (IS), intra-daily variability (IV), and falling asleep time (FAT).

The SE was the duration of sleep expressed as a percentage of time in bed. TST was the amount of sleep achieved in 24-h sleep episodes; this time was equal to the total sleep episode less the periods of wakefulness.

Both IS and IV were deemed to be the measures of the strength of circadian rhythmicity. The MotionWare software provided the function named non-parametric circadian rhythm analysis (NPCRA). The NPCRA was based on the work of Dr. Eus Van Someren at the Netherlands Institute for Brain Research (Van Someren et al., 1999). IS reflected the degree of consistency of activity patterns from one day to the next; values ranged from 0 to 1; and the closer the value was 1, the greater the stability. IV reflected the fragmentation of the rest-activity rhythm; that is, the rate of shift between rest and activity. High IV may have indicated daytime napping and/or frequent nighttime arousals; values ranged from 0 to 2, and the closer the value was 2, the greater the degree of fragmentation.

The FAT was defined as the time when the subject first fell asleep. The 7-day mean values of FAT were considered to determine the phase-angle DLMO-FAT, expressed in minutes. The phase angle DLMO-bedtime could be calculated through a combination of the actigraphic findings with information derived from the 24-h sleep/wake diaries that the patients completed.

The 7-day mean values of SE and TST were considered as the primary outcomes of the study because they were more reliable than the questionnaires in the detection of variations in sleep parameters.

#### Subjective Sleep Parameters

Two questionnaires, which were presented in a validated version of the Italian language, were administered before and after the 4-week light therapy to assess the subjective measures of nocturnal sleep quality and daytime sleepiness. The PSQI and ESS data represented the secondary outcomes of our study. The PSQI is a tool to measure sleep quality in clinical populations. It comprises 19 items that generate 7 component scores (i.e., sleep quality, sleep latency, sleep duration, habitual SE, sleep disturbances, use of sleep medication, and daytime dysfunction). The sum of the component scores yields a global score. Global scores >5 points indicate poor sleep quality (Curcio et al., 2013).

The ESS is an eight-item assessment of daytime sleepiness, with possible scores of 0–24. A score of over 10 is considered indicative of an abnormal degree of daytime sleepiness (Vignatelli et al., 2003).

#### Neuropsychological Assessment

Neuropsychologists at the Neuropsychology/Alzheimer’s Disease Assessment Unit administered neuropsychological tests at the beginning of the protocol and within a week after light delivery. The MMSE is a screen that is widely used to assess the levels of cognitive impairment. The study provided MMSEc scores as a secondary outcome. In addition, patients were measured according to the NPI in order to assess the most typical behavioral disturbances that occur in dementia patients (i.e., delusions, hallucinations, dysphoria, anxiety, agitation/aggression, euphoria, disinhibition, irritability/lability, apathy, and aberrant motor activity).

#### Statistical Analysis

The Statistical Package for the Social Sciences (SPSS) for Windows, version 21.0, was used to perform the statistical analysis. Primary and secondary outcomes were tested within each cohort of patients (active and sham light treatments) through a comparison of pre- and post-treatment variables. The same analysis was performed for the subgroups of both ECPpts and LCPpts in each cohort of patients.

Variability was analyzed despite the limitation of the small sample size. To account for variability, the researchers focused on the univariate analyses of the outcome measures within the active light treatment and sham groups before and after light treatment. To consider the continuous variables that made up the primary and secondary outcomes, pre- and post-treatment measures were compared within groups through the use of a non-parametric test, i.e., the Wilcoxon signed-rank test. For dichotomous variables, the McNemar test was performed.

Due to the extremely small sizes of the subgroups of both ECPpts and LCPpts, only descriptive statistics were performed on these cohorts.

Continuous variables were expressed as median and IQRs (quartile 1–quartile 3), with the exception of DLMO values that are also reported as mean value ± SD. In terms of normality, median and IQRs of the differences between pre- and post-treatment observations (pre–post) for each outcome measure were also reported. The level of significance was set at 0.05.

## Results

### Effects of Light Protocol on Circadian Parameters

#### Effects on Dim Light Melatonin Onset Time

**FIGURE 3:**
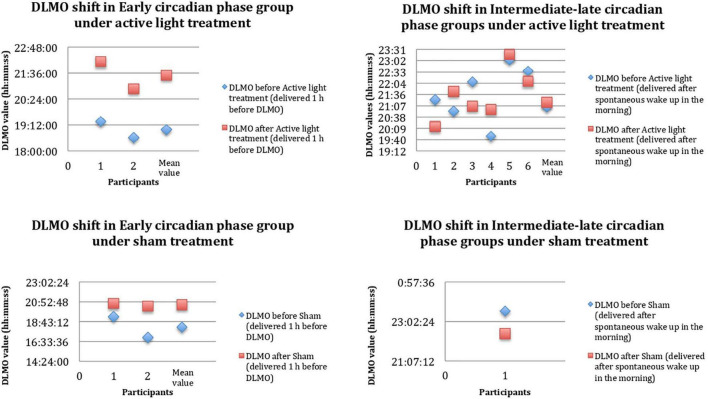
DLMO shift before (PRE, blue rhombuses) and after (POST, red squares) 4-week Luminette protocol. The *x*-axis shows any single patient in progressive numbers, and the *y*-axis shows the hours of the day. DLMO, dim light melatonin onset. After a single-blind 4-week tailored light therapy, patients with AD presented a circadian phase shift toward a later chronotype (mean values). These data are probably the result of a more consistent circadian phase shift in the Early Circadian Phase patients (ECPpts) compared to Late Circadian Phase patients (LCPpts).

Considering the AD group as a whole, the measurements of salivary melatonin showed a mean baseline DLMO of 21:18 ± 1:34. This indicated an average intermediate circadian phase. The DLMO values indicated evening profiles in about 46% of the subjects. At the individual level, the lowest significant value of DLMO shift after active or sham light treatment was set at 20 min. In two members of the sham group, the DLMO after sham light treatment could not be calculated due to the irregularities of the secretion curves (“bizarre curves”). Sham light treatment was associated with DLMO shifts in two of the three patients with evaluable DLMOs (Figure 3). In the active light treatment group, a phase shift occurred in seven out of eight patients, as shown in Figure 3. The mean shift was 84.14 ± 49.4 min. In the active light treatment group, the direction of the shift varied with the timing of Luminette exposure in five patients; the DLMO advanced in the case of exposure to light after the initial DLMO time, and it was delayed in the case of exposure to light before the initial DLMO time (Figure 3). In two intermediate circadian phase patients, an unexpected delay in DLMO was found, whereas one late circadian phase patient did not show a significant DLMO shift (Figure 3). The phase shift was wider, but not significantly, in the subgroup of ECPpts than in the subgroup of LCPpts, as listed in Table 2 and displayed in Figure 3.

**TABLE 2:** Primary and secondary outcomes, and other circadian parameters about the subgroups of both Early Circadian Phase patients (ECPpts) and Late Circadian Phase patients (LCPpts) before (PRE) and after (POST) active light protocol.

	ECPpts active light protocol				LCPpts active light protocol			
	PRE	POST	Median change	IQR (delta change)	PRE	POST	Median change	IQR (delta change)
Number of pts	2	2			3	3		
DLMO (hh:mm) (median, IQR)	18:59 (18:48–19:10)	21:30 (21:11–21:49)			22:36 (22:22–22:50)	22:15 (21:40–22:46)		
Phase angle DLMO-bedtime (minutes; median, IQR)	135.50 (116.75–154.25)	−7.50 (−14.75 to −0.25)			54.00 (39.50–82.50)	45.00 (13.50–109.50)		
Phase angle DLMO-FAT (minutes; median, IQR)	152.50 (134.25–170.75)	18.00 (−9.50 to 45.50)	134.5	116–153	110.00 (86.50–137.50)	88.00 (65.50–121.50)	20	10–22
% Patients with phase angle DLMO-bedtime <40 min	0%	100%			33.3%	33.3%		
% Patients with phase angle DLMO-FAT <40 min	0%	50%			0%	0%		
% Patients with phase angle DLMO-bedtime <60 min	0%	100%			66.7%	66.7%		
% Patients with phase angle DLMO-FAT <60 min	0%	50%			0%	33.3%		
% Patients with phase angle DLMO-bedtime >90 min	100%	0%			33.3%	33.3%		
% Patients with phase angle DLMO-FAT >90 min	100%	0%			66.7%	33.3%		
Sleep efficiency (%) (median, IQR)	79.75 (79.38–80.13)	77.75 (76.62–78.87)	2	−1 to 5	81.60 (80.95–82.40)	74.10 (74.00–80.45)	6.2	−3.6 to 7.7
24 h total sleep time (minutes; median, IQR)	549.75 (529.51–544.69)	607.17 (543.62–670.73)	−57.43	−144.07 to 29.21	485.29 (453.15–508.43)	466.7 (437.49–525.31)	12.71	−52.35 to 18.59
Interdaily stability (median, IQR)	0.64 (0.64–0.65)	0.60 (0.57–0.63)	0.04	−0.03 to 0.11	0.62 (0.57–0.68)	0.67 (0.57–0.70)	0.025	−0.05 to 0.06
Intradaily variability (median, IQR)	1.07 (0.98–1.16)	1.22 (1.06–1.38)	−0.145	−0.29 to 0.1	0.91 (0.81–1.03)	0.92 (0.89–0.97)	−0.197	−0.1 to 0.1
PSQI (mean score (median, IQR)	7 (7–8)	2 (2–2)	6	6–6	5 (5–6)	5 (3–5)	1	0–2
ESS (median, IQR)	7 (4–11)	2 (1–3)	4.5	−4 to 13	5 (3–5)	4 (2–5)	0	−21
MMSEc (median, IQR)	18.20 (16.10–20.30)	22.55 (22.27–22.82)	−4.35	−8 to −0.7	21.10 (19.40–21.15)	22.70 (21.90–22.95)	−2	−5 to 0

*Number of pts, number of participants; ECPpts, Early Circadian Phase patients; LCPpts, Late Circadian Phase patients; IQR, interquartile range (quartile 1–quartile 3); DLMO, dim light melatonin onset; FAT, falling asleep time at the actigraphy recording; PSQI, Pittsburgh Sleep Quality Index; ESS, Epworth Sleepiness Scale; MMSEc, Mini-Mental State Examination adjusted for age and educational levels.*

#### Effects on Phase Angles (Dim Light Melatonin Onset-Falling Asleep Time and Dim Light Melatonin Onset-Bedtime)

The phase angle for DLMO-bedtime was longer than 1 h at the baseline in 62.5% of patients with AD in the active light group and 67.7% of those in the sham group (Table 3).

**TABLE 3:** Primary and secondary outcomes, and other circadian parameters before (PRE) and after (POST) active light/sham protocol.

	AD active light protocol					Sham protocol				
	PRE	POST	Median change	IQR (delta change)	*P*-value	PRE	POST	Median change	IQR (delta change)	*P*-value
Number of pts	8	8				5 (3 pts for DLMO and related measures)	5 (3 pts for DLMO and related measures)			
DLMO (hh:mm) (median, IQR)	21:08 (19:43–22:15)	21:25 (20:56–22:09)				19:15 (18:07–21:24)	20:44 (20:34–21:35)			
Phase angle DLMO-bedtime (minutes; median, IQR)	104.5 (56.25–137.75)	30.50 (0.75–65.00)				195.00 (111.00–202.50)	136.00 (115.50–159.50)			
Phase angle DLMO-FAT (minutes; median, IQR)	113.00 (89.25–167.50)	80.50 (34.50–92.75)	51	15–100.5	0.023	232.00 (197.00–312.50)	154.00 (123.50–157.00)	102	72–196	0.0625
% Patients with phase angle DLMO-bedtime <40 min	12.5%	50%				33.3%	0%			
% Patients with phase angle DLMO-FAT <40 min	0%	25%				0%	0%			
% Patients with phase angle DLMO-bedtime <60 min	37.5%	75%				33.3%	0%			
% Patients with phase angle DLMO-FAT <60 min	0%	37.5%				0%	0%			
% Patients with phase angle DLMO-bedtime >90 min	62.5%	25%				66.6%	100%			
% Patients with phase angle DLMO-FAT >90 min	75%	25%				100%	100%			
Sleep efficiency (%) (median, IQR)	81.05 (79.98–83.75)	79.95 (75.15–81.52)	2.9	−2.3 to 6.95	0.328	86.00 (78.80–89.10)	85.95 (78.00–88.30)	0.15	− 4.4 to 5	0.875
24 h total sleep time (minutes; median, IQR)	506.79 (473.68–546.23)	519.75 (476.72–586.36)	3.5	−71.49 to 18.37	0.64	423.85 (381.07–447.64)	487.99 (419.82–528.69)	−11.34	−52.25 to 38.05	0.87
Interdaily stability (median, IQR)	0.62 (0.57–0.67)	0.67 (0.63–0.70)	−0.04	−0.07 to 0.05	0.54	0.67 (0.60–0.77)	0.63 (0.56–0.68)	0.0185	−0.04 to 0.1	0.625
Intradaily variability (median, IQR)	0.82 (0.73–0.97)	0.91 (0.86–0.97)	−0.146	−1.45 to 0.09	0.41	0.82 (0.80–0.82)	0.75 (0.66–0.89)	0.05	−0.05 to 0.4	0.625
PSQI (mean score (median, IQR)	6 (4–7)	2 (2–4)	1	0–5	0.06	3 (3–4)	4 (2–5)	0	−2 to 1	0.62
ESS (median, IQR)	4 (1–6)	2 (1–4)	0.5	−1.5 to 2.5	0.67	3 (2–4)	3 (3–4)	0	−2 to 0	0.5
MMSEc (median, IQR)	19.20 (17.63–21.13)	21.55 (20.12–22.80)	−0.85	−3.5 to 0.35	0.03	26.00 (20.40–27.10)	23.40 (22.40–24.30)	1	−2 to 1.7	1

*Number of pts, number of participants; IQR, interquartile range (quartile 1–quartile 3); DLMO, dim light melatonin onset; FAT, falling asleep time at the actigraphy recording; PSQI, Pittsburgh Sleep Quality Index; ESS, Epworth Sleepiness Scale; MMSEc, Mini-Mental State Examination adjusted for age and educational levels.*

Conversely, the baseline phase angle for DLMO-FAT was longer than 1 h in all patients in both groups (Table 3).

These phase angles were significantly reduced after active light treatment (*p* = 0.023), with 50% of patients who received active light treatment showing a DLMO-FAT phase angle between 40 and 90 min after the treatment (Table 3).

The subgroups of both ECPpts and LCPpts showed the same trend in terms of decreases in the duration of the DLMO-FAT phase angles, with deeper reductions in the ECP subgroup after light treatment (Table 2). Furthermore, 50% of ECPpts and 66.7% of LCPpts who underwent active light treatment reached DLMO-FAT phase angles between 40 and 90 min after the treatment (Table 2).

### Primary Outcomes

Both SE and 24-h TST did not improve in either the active or the sham light treatment groups after the 4-week treatment protocol (Table 3). The same result was found when the subgroups of both ECPpts and LCPpts were taken into account (Table 2).

The IS and the IV were unchanged before and after light therapy (Tables 2, 3).

### Secondary Outcomes

Subjective sleep quality improved in the active light treatment group, showing a positive trend that was not observed in the sham group (*p* = 0.62) (Table 3). This improvement was greater in the subgroup of ECPpts than in the subgroup of LCPpts (Table 2).

Sleepiness, evaluated by means of the ESS, did not show any significant variation after treatment in either group (Table 3).

The MMSEc mean score significantly improved in the active treatment group (*p* = 0.03), while it did not significantly change in the sham group (*p* = 1) (Table 3). At the individual level, the lowest significant value of MMSEc shift was set at 2 points. Individual MMSEc scores improved in six of eight patients with AD, who received active light treatment, while the other two showed stable scores. MMSEc scores worsened in three of the five patients with AD who received sham treatment (Table 3).

In the active light treatment group, we found a greater, but insignificant, improvement among the ECPpts that was not observed in the LCPpts (Table 2).

Beck’s Depression Inventory and the NPIs did not alter after light therapy in either group.

## Discussion

Our data show that light treatment had no meaningful effect on nocturnal SE and 24-h TST. These results are in agreement with those suggested by other research in the literature (Mishima et al., 1994; Lyketsos et al., 1999).

These findings are at odds with the literature results that have indicated a substantial improvement in actigraphy parameters, such as SE, IS, and IV, after light delivery (Van Someren et al., 1997; Ancoli-Israel et al., 2003; Fetveit et al., 2003).

In our study, light therapy also had a beneficial effect on subjective sleep quality, as documented by the PSQI score. Our results are in keeping with the literature data (Sloane et al., 2015; Figueiro et al., 2019), which indicate that light exposure in AD is associated with an improvement of PSQI scores. These authors also found a beneficial effect of light on the levels of depression and agitated behavior. In our study, the Beck scores did not change after active light treatment. However, no patient in our sample suffered major depression, and neither patients nor caregivers reported agitated behaviors, probably because the participants were at an early stage of the disease.

Given the limited sample size in this study, our results concerning sleep parameters may have been influenced by one patient, who showed noticeable results. This patient showed a robust increase in rest-activity rhythm consolidation; TST increased from 590 to 734 min; SE remained stable; and daily sleepiness and PSQI scores were strongly improved. This patient also showed an MMSEc score improvement of 8 points (from 14 to 22) after 4 weeks of light treatment. Such a great improvement is remarkable in a neurodegenerative form of dementia but must be considered cautiously because MMSE scores are known to fluctuate over time.

Cognitive performance improved significantly in the patients with AD, who were exposed to active light treatment (*p* = 0.03), while it worsened in the patients who received sham treatment. The improvement we observed was probably due to the early stage of AD in our patients. Yamadera et al. (2000) reported a more beneficial effect of bright light on MMSE scores in early than in advanced forms of AD. These researchers attributed these data to the reduced damage that is observed in the retinogeniculate pathway and the suprachiasmatic nucleus in early compared with advanced forms of AD. However, we cannot rule out the possibility that the cognitive improvement we observed may have been influenced by the results for one patient, who presented a remarkable improvement in MMSEc.

The light protocol we used proved to be efficacious in shifting the circadian phase in patients with AD congruently with the phase response curve of melatonin to light exposure. It also reset the DLMO-bedtime and DLMO-FAT phase angles, which meant that the patients went to bed earlier after DLMO and fell asleep more quickly than they had before the treatment.

It is difficult to explain the shifts of DLMO that were observed in two patients in the sham group and in the two intermediate patients in the active group who had delays in DLMO (shifts to the opposite than expected direction).

These patients were not reported to have been exposed to confounders such as inappropriate light delivery or changes in their sleep habits. This finding could be interpreted as spontaneous fluctuations in melatonin secretion. However, inappropriate light delivery could not be ruled out for certain as we did not control for lighting conditions in the domestic environment.

We found improvements in both subjective impressions of sleep quality and MMSEc scores in the subgroup of ECPpts compared with the LCPpts after active light treatment. The subgroup of ECPpts also showed larger phase angle shifts than the LCPpts. It is known that light may be beneficial for sleep and cognitive functioning through its effects on the circadian system (Figueiro et al., 2019). It is both intriguing and hazardous to attribute the improvement of subjective sleep quality and MMSEc scores to the effects of light on the circadian systems of our patients. The sample size was small, and there was no evidence of a cause-effect relationship between light-induced circadian effects and the improvement of sleep and cognitive functioning in our patients. However, these results should be investigated in detail in future studies that employ bigger sample sizes.

It would be of particular interest to confirm an association between extreme DLMO-FAT phase angles, especially those that are longer than 2 h, and reduced sleep quality and impaired cognitive functioning. It would also be interesting to discover whether a light-induced reset of these parameters is associated with the beneficial effects on sleep and cognitive functioning in patients with AD.

It would also be worthwhile to confirm whether patients with AD with an ECP are more prone to undergo a light-induced phase shift that aligns with benefits in terms of sleep quality and cognitive performance than those with a late circadian phase.

A favorable aspect of our protocol was its good feasibility. There was minimal risk of harm to the participants, as there have been no known safety risks associated with the device that was used except for modest and transient eye disorders (i.e., conjunctival reddening, eye irritation, mild headache, and eye fatigue), for which the patients were screened before and through the study (Botanov and Ilardi, 2013). Most of the patients were able to perform the melatonin test and complete it correctly. Luminette glasses proved to be suitable for the study and easy to use, with only one patient dropping out because of mild side effects due to light exposure (ocular irritation and burning). As we enrolled patients with mild-to-moderate AD, we could administer the questionnaires directly to the participants, which avoided the need to use caregivers as intermediates. Proxy data are not reliable in the provision of subjective perceptions of sleep quality.

### Limitations of the Study

The results of our study may have been influenced by the size of the sample and the predominance of males among the participants (M/F: 9/4). The sex-related impacts on the efficacy of light exposure have been reported in AD (Jee et al., 2020); specifically, female patients show major improvements in sleep quality and mood after such treatment. Future studies of new therapeutics for sleep disorders in AD should stratify the results according to sex/gender.

We did not control for lighting conditions in the domestic environment. This omission represents an important limitation of the study because we could not evaluate the “light diet” of our subjects during the 4-week protocol. In contrast, Figueiro et al. (2019) focused on the environment in which the study subjects lived by arranging appropriate lighting conditions that were tailored to the maximal entrainment of the circadian system (Figueiro et al., 2019). The development of a “light diary” would be a helpful tool for the assessment and/or modification of the daily/nightly light exposure of participants in future studies.

## Conclusion

This RCT offers promising, applicable results. It should be replicated through the use of a larger sample size and perhaps a longer duration of treatment (e.g., 6 months).

## Data Availability Statement

The raw data supporting the conclusions of this article will be made available by the authors, without undue reservation.

## Ethics Statement

The studies involving human participants were reviewed and approved by IRCCS Mondino Foundation Ethics Committee. The patients/participants provided their written informed consent to participate in this study.

## Author Contributions

RC: conceptualization, data curation, formal analysis, investigation, methodology, project administration, visualization, and writing – review and editing. DS and GG: data curation, formal analysis, investigation, methodology, and writing – review and editing. StC and EP: formal analysis, investigation, and methodology. FR and FB: data curation, formal analysis, investigation, and methodology. CG, SiC, MP, and SB: data curation, formal analysis, and investigation. ES, MT, LP, and AM: resources, supervision, and writing – review and editing. RM: conceptualization, methodology, project administration, resources, supervision, visualization, and writing – review and editing. All authors contributed to the article and approved the submitted version.

## Conflict of Interest

The authors declare that the research was conducted in the absence of any commercial or financial relationships that could be construed as a potential conflict of interest.

## Publisher’s Note

All claims expressed in this article are solely those of the authors and do not necessarily represent those of their affiliated organizations, or those of the publisher, the editors and the reviewers. Any product that may be evaluated in this article, or claim that may be made by its manufacturer, is not guaranteed or endorsed by the publisher.

## Footnotes

1
www.openepi.com
